# Effects of Carbolic Acid on the Economy, on Vegetable Parasites, and Diseases of the Skin

**Published:** 1869-11

**Authors:** 


### Effects of Carbolic Acid on the Economy, on Vegetable Parasites, and Diseases of the Skin.

Dr. Neumann, of Vienna, has published, in Dr. Pick's *Archiv fur Dermatologie und Syphilis,* Part III. (1869,) an excellent article on the above subject. The author experimented largely on animals and plants, and has used the acid in a certain number of cases, the principal of which he relates. Dr. Neumann sums up as follows: Carbolic acid is an energetic poison, which acts directly on the nervous system; its external or internal use may cause death. It acts three times more quickly when injected under the skin than when taken into the stomach. The acid is useful in scaly skin diseases, but especially in their early stage; it may be used as a caustic in chronic infiammations, and in parasitic affections. The acid, finally, possesses the power of arresting the germination of the lower vegetable organisms; but the solutions must for this purpose be stronger than has been advised—viz., 1 in 500 or 300, and not 1 in 1000.—*Lancet,* Sept. 18, 1869.—*Med. News.*

